# Mechanical, physical and thermal properties of composite materials produced with the basidiomycete *Fomes fomentarius*

**DOI:** 10.1186/s40694-023-00169-8

**Published:** 2023-12-04

**Authors:** Bertram Schmidt, Carsten Freidank-Pohl, Justus Zillessen, Lisa Stelzer, Tamara Núñez Guitar, Carsten Lühr, Henri Müller, Fangxing Zhang, Jörg U. Hammel, Heiko Briesen, Sascha Jung, Hans-Jörg Gusovius, Vera Meyer

**Affiliations:** 1 Chair of Applied and Molecular Microbiology, Technische Universität Berlin, Str. des 17. Juni 135, 10623 Berlin, Germany; 2 Department Systems Process Engineering, Leibniz-Institute for Agricultural Engineering and Bioeconomy (ATB), Max-Eyth-Allee 100, 14469 Potsdam, Germany; 3 School of Life Sciences Weihenstephan, Chair of Process Systems Engineering, Technical University of Munich, 85354 Freising, Germany; 4 Institute of Materials Physics, Helmholtz-Zentrum Hereon, Max-Planck-Str 1, 21502 Geesthacht, Germany

**Keywords:** *Fomes fomentarius*, Fungal-based composite material, Circular economy, Bioeconomy, Hemp, Composite material, Mycelium-based material, Compressive strength, Flexural strength, Tensile strength, Flammability, Insulation

## Abstract

**Background:**

To achieve climate neutrality, fundamentally new concepts of circularity need to be implemented by the building sector as it contributes to 40% of anthropogenic CO_2_ emission. Fungal biotechnology can make a significant contribution here and help eliminate fossil dependency for building material production. Recently, we have shown that the medicinal polypore *Fomes fomentarius* feeds well on renewable lignocellulosic biomass and produces composite materials that could potentially replace fossil fuel-based expanded polystyrene as insulation material.

**Results:**

In this study, we explored the mechanical, physical, and thermal properties of *F. fomentarius*-based composite materials in more detail and determined key performance parameters that are important to evaluate the usability of *F. fomentarius*-based composite materials in the construction sector. These parameters were determined according to European standards and included compressive strength, modulus of elasticity, thermal conductivity, water vapour permeability, and flammability of uncompressed composites as well as flexural strength, transverse tensile strength, and water absorption capacity of heat-pressed composites, among others. We could show that uncompressed composites obtained from *F. fomentarius* and hemp shives display a thermal conductivity of 0.044 W (m K)^−1^ which is in the range of natural organic fibres. A water vapour permeability of 1.72 and classification into flammability class B1 clearly surpasses fossil-based insulation materials including expanded polystyrene and polyurethane. We could furthermore show that heat-pressing can be used to reliably generate stiff and firm particleboards that have the potential to replace current wood-based particleboards that contain synthetic additives. X-ray microcomputed tomography finally visualized for the first time the growth of hyphae of *F. fomentarius* on and into the hemp shive substrates and generated high-resolution images of the microstructure of *F. fomentarius*-based composites.

**Conclusion:**

This study demonstrates that fungal-based composites produced with *F. fomentarius* partially meet or even exceed key performance parameters of currently used fossil fuel-based insulation materials and can also be used to replace particleboards.

**Supplementary Information:**

The online version contains supplementary material available at 10.1186/s40694-023-00169-8.

## Introduction

Recently, fungal derived biomaterials have emerged as potential sustainable alternatives for petroleum-based packaging, textiles, and construction material [1, 2]. Crucially, the fungal kingdom is unique in the ability to degrade lignocellulosic substrates, and thus constitutes the only solution to recycle megatons of global agricultural and forestry residual waste streams into renewable materials. Thus, fungal biomaterials are predicted to play a vital role in developing a sustainable bioeconomy and can help achieve the United Nations development goals of sustainable communities, responsible consumption, and the mitigation of climate change [3, 4].

Mainly species of the genera *Ganoderma*, *Pleurotus* and *Trametes* are in the current focus of research to generate a variety of pure mycelium or fungal-based composite materials to be harnessed as packaging material, thermal insulation, acoustic insulation, construction material, as well as leather. For an overview on the production and properties of biomaterials achieved by these fungal species, the reader is directed to recent reviews which have elegantly summarized the current state-of-the-art [1, 2, 5, 6].

An outstanding species in the fungal biomaterial revolution is the common terrestrial white-rot polypore *Fomes fomentarius* also known as tinder or ‘amadou’ fungus. In its natural niche, it infects sick or dead trees, e.g. hardwoods including birches and beeches and softwoods including conifers, whereby its hyphae penetrate damaged bark, ultimately leading to colonization and degradation of the plant tissue, and production of large perennial fruiting bodies which are very stable, light-weighted and water-repellent [7, 8]. Even five thousand years ago, fruiting bodies of *F. fomentarius* were used to carry ember as reported by archaeological findings of the Iceman’s mummy ‘Ötzi’ [9]. Various references describe a rich and millennia-old tradition in the medicinal (e.g. treatment against cancer, bladder disorders, use as diuretic, laxative and tonic for nerves, use for wound healing and as warming compresses) and spiritual use of *F. fomentarius* fruiting bodies in very diverse regions ranging from Japan, China, India to Europe [9].

Bioprospecting of local wood ecosystems by our group clearly identified *F. fomentarius* as an ideal species for renewable biomaterial generation due to robust laboratory growth on a wide range of lignocellulosic plant residues (e.g., hemp shives, rapeseed straw, poplar sawdust) [10]. We thus developed this fungus as cell factory for the production of fungal-plant composite materials, which included optimizing growth media and developing a laboratory manufacturing process, and could demonstrate that composites based on hemp shives and rapeseed straw obtained with *F. fomentarius* have a compressive strength in the range of petroleum-based expanded polystyrene (EPS) [10]. A first life cycle assessment has demonstrated that building bricks consisting of composite materials produced by *F. fomentarius* with hemp shives, rapeseed straw or poplar sawdust are superior in the categories ‘climate change’, ‘smog’ and ‘water scarcity’ when compared to building blocks consisting of concrete or limestone [11]. As additive manufacturing (3D-printing) of buildings is a rapidly evolving field that promises on-site construction of buildings with increased resource-use efficiency and with nearly no wastage of raw construction materials, we also recently provided proof-of-concept that mycelia of *F. fomentarius* can be 3D-printed and generate composites with compressive strength comparable to that of EPS [12].

Cultivation of *F. fomentarius* on hemp shives is our main interest for the generation of fungal-based composite materials. Hemp is experiencing a renaissance as environmentally friendly source for natural fabrics to replace synthetic fibres and cotton, because hemp cultivation ensures high yields even with very low water and nutritional requirements and without the use of pesticides or herbicides [13]. The global market for hemp and hemp derived products is estimated to have US$ 4.3 billion by 2022 and was projected to grow at a significant CAGR of 10.5% during 2022–2030 [14]. In Germany, hemp cultivation has more than doubled over the past five years [15]. At present, it is mainly the fibres of the hemp plant that are used in technical or clothing products, while applications with higher added value are still being sought for shives as the second main product with about 50% by mass. Thus, with a composition of about 34–46% w/w cellulose, 25–37% w/w hemicellulose and 19–28% w/w lignin [16, 17], hemp shives are ideal substrates for white-rot basidiomycetes like *F. fomentarius*.

In the current study, we explored the mechanical, physical, and thermal properties of composite materials obtained from *F. fomentarius* and hemp shives in more detail. We determined compressive strength, thermal conductivity, water vapour permeability and flammability of composites according to European standards (EN standards) to evaluate their application potential as future insulation material in the building sector. We furthermore applied heat-pressing as post-processing treatment of *F. fomentarius*-hemp composites to generate natural fibre boards as potential alternatives for particleboards and medium density fibre (MDF) boards. These boards were examined regarding water absorption, flexural strength, and transverse tensile strength according to EN standards. Comparative scanning electron microscopic (SEM) and X-ray micro-computed tomography (µCT) analyses of unpressed and pressed composites allowed us to gain morphological insights into the growth of *F. fomentarius* on hemp shives and to determine changes in properties due to heat-pressing.

## Results and discussion

### *F. fomentarius* composites as potential insulation material

**Fig. 1:**
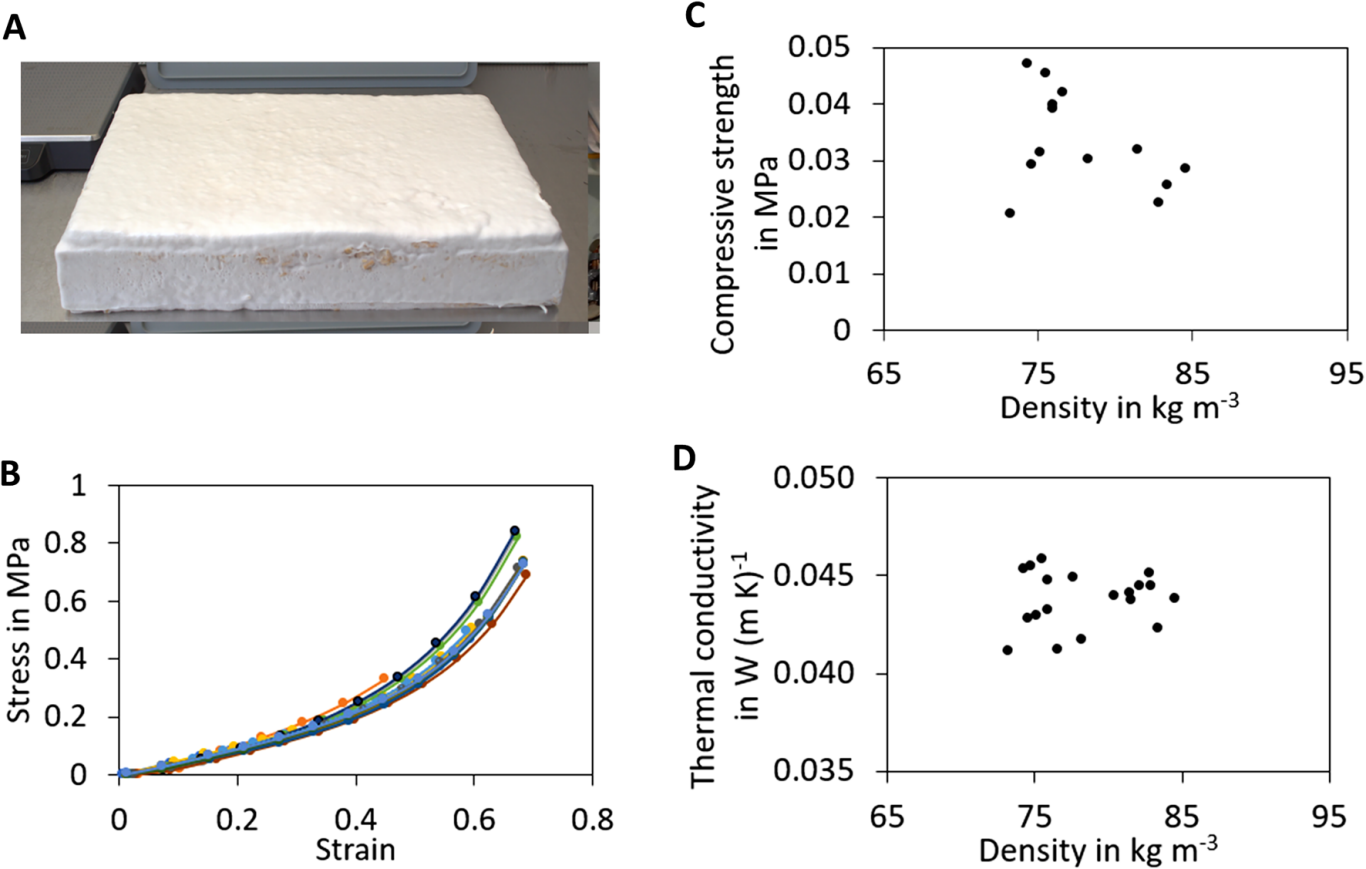
Compressive strength and thermal conductivity testing of fungal-based composite made of *F. fomentarius* and hemp shives. **A** Image of an exemplar specimen used for compressive and conductivity testing. **B** Stress–strain curves for all specimens with different height. **C** Compressive strength at 10% compression (δ_10_) as a function of the density of all specimens. **D** Thermal conductivity values determined for all specimens with different height

In order to test the suitability of composites obtained from *F. fomentarius* and hemp shives for application in the building sector, the compressive strength was determined on cuboid composite specimens of different heights (5, 6, 8 and 10 cm, respectively) with at least four biological replicates per height (Fig. 1). These heights were chosen as they are frequently used for EPS plates within the building sector. As the laboratory manufacturing process is still performed manually and high variability of properties is an inherent characteristics of all biological materials (e.g. heterogeneous hemp shives varying in form and size, heterogeneous growth of *F. fomentarius* around and into hemp shives), the density of all specimens were recorded. As depicted in Fig. 1, the densities of the fungal composites varied in the range of 78.39 ± 4.53 kg m^−3^ and the stress–strain relationship thus scatter to a certain extent (Fig. 1B). The difference is smaller at the very beginning (which is characterized by deformation of the aerial mycelium that surrounds all composites) but increases during compression. The compressive strength σ_10_ at 10% strain is 0.020–0.047 MPa (Fig. 1C), which is rather low but confirms earlier studies on fungal-based composites based on *Ganoderma*, *Pleurotus* and *Trametes* spp. which also display limited load-bearing capacities [2, 18, 19]. However, such low compressive strengths would not prevent the use of fungal-based composites as interior insulation, as the composites would be inserted between beams that ensure stability. However, for use as external insulation, a compressive strength of 0.10–0.15 MPa at 10% compression is specified for insulation materials [20]. Interestingly, the thermal conductivity of *F. fomentarius* composites varied between 0.0411 and 0.0458 W (m K)^−1^ (Fig. 1D). The calculated mean value of 0.044 W (m K)^−1^ is about 30% higher than the thermal conductivity of EPS with 0.035 W (m K)^−1^. In other words, an EPS insulation panel with a thickness of 6 cm would have to be replaced with a fungal-based panel with a thickness of 8 cm to ensure the same insulation effect.

Further parameters that are relevant for the assessment of insulation materials are water vapour emission and flammability. To assess these for *F. fomentarius* composites, we selected one exemplar specimen from each to perform respective testing according to EN standards (see Materials and Methods). For a specimen of 5 cm height, the vapour diffusion current density g was calculated as 19.65 g (m^2^ h)^−1^, the vapour diffusion transmittance W as 8.22 mg (m^2^ h Pa)^−1^, and the vapour diffusion conductivity coefficient δ as 0.411 mg (m h Pa)^−1^. The dimensionless vapour diffusion resistance coefficient µ was calculated as 1.72, indicating that composites made of *F. fomentarius* and hemp shives can be classified as vapour-diffusible with a µ value comparable to other natural plant-based insulation materials [20].

**Fig. 2:**
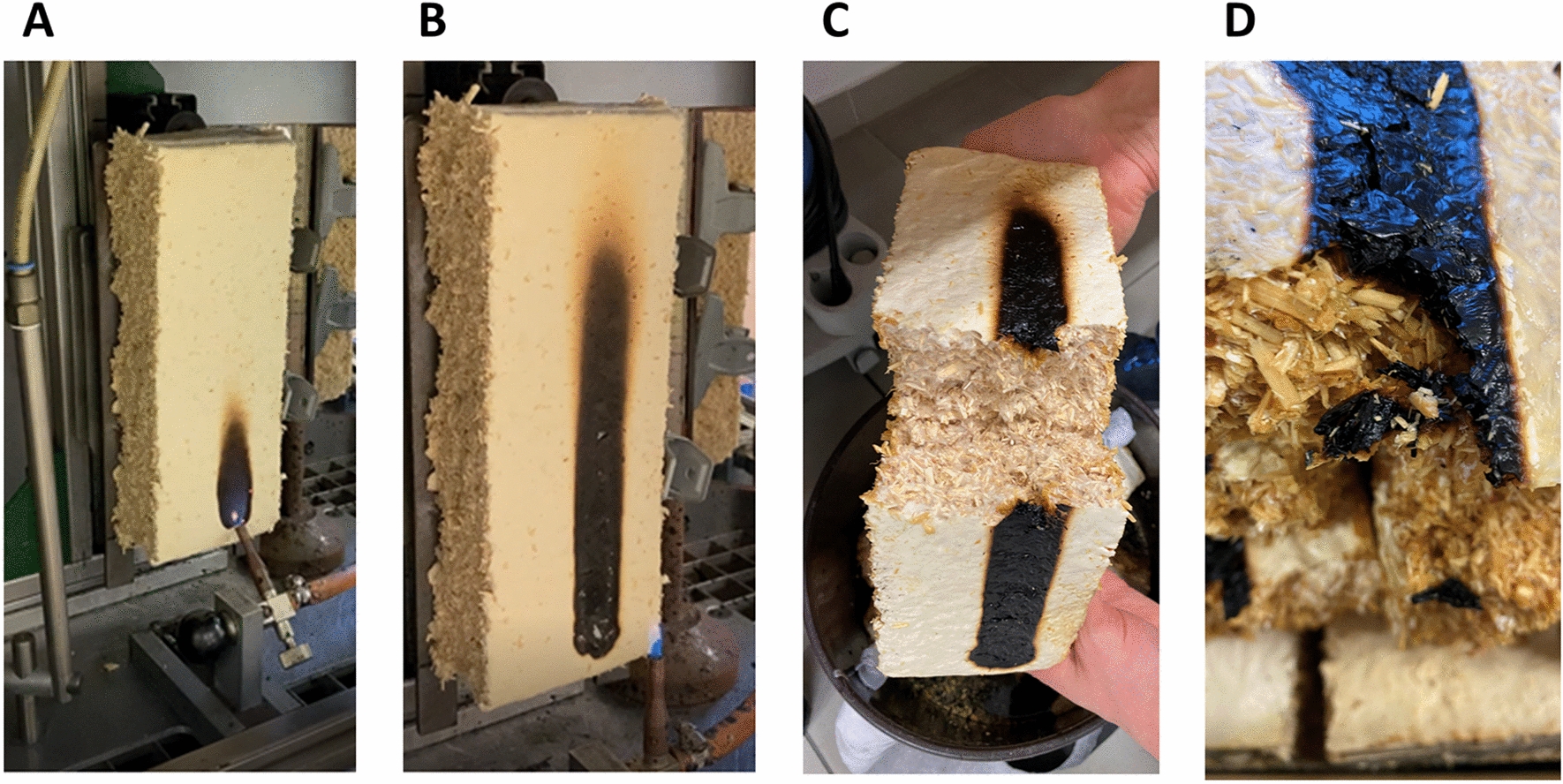
Flammability testing of an exemplar fungal-based composite made of *F. fomentarius* and hemp shives. **A**, **B** Specimen at the test facility at 1 s (**A**) and 30 s (**B**) after ignition. **C**, **D** Burning did not reach deeper layers of the composite and took place only on the surface

Various flaming times are used according to EN standards to specify different flammability classes: B/C/D (low to medium flammable; 30 s) and E (highly flammable; 15 s). Non-flammable materials such as concrete, glass or steel are classified into the flammability class A. We thus tested one scenario in which the flame was ignited at a small location for 30 s. During this period, fire expansion was only observed perpendicular to the flame (Fig. 2) but not horizontally as usually observed with highly flammable materials such as EPS (class E). As the perpendicular flame formation did not exceed a length of 150 mm above the flame point 60 s after the start of the test and did also not extend into the composite (Fig. 2B–D), the material was classified as B1 according to EN 13501–1. No burning parts fell off either and smoke development was only moderate. Thus, composites of *F. fomentarius* and hemp shives were classified as B1-s2-d0. However, future analyses should address the composition of the smoke components in more detail.

### *F. fomentarius* heat-pressed composites as potential particleboard material

In order to increase the load-bearing capacities of composites made of *F. fomentarius* and hemp shives, we tested heat-pressing as post-processing method after drying the composites, an approach which has already successfully been used to increase the mechanical strength of fungal-based composites made of *Pleurotus* and *Trametes* species [18].

**Fig. 3:**
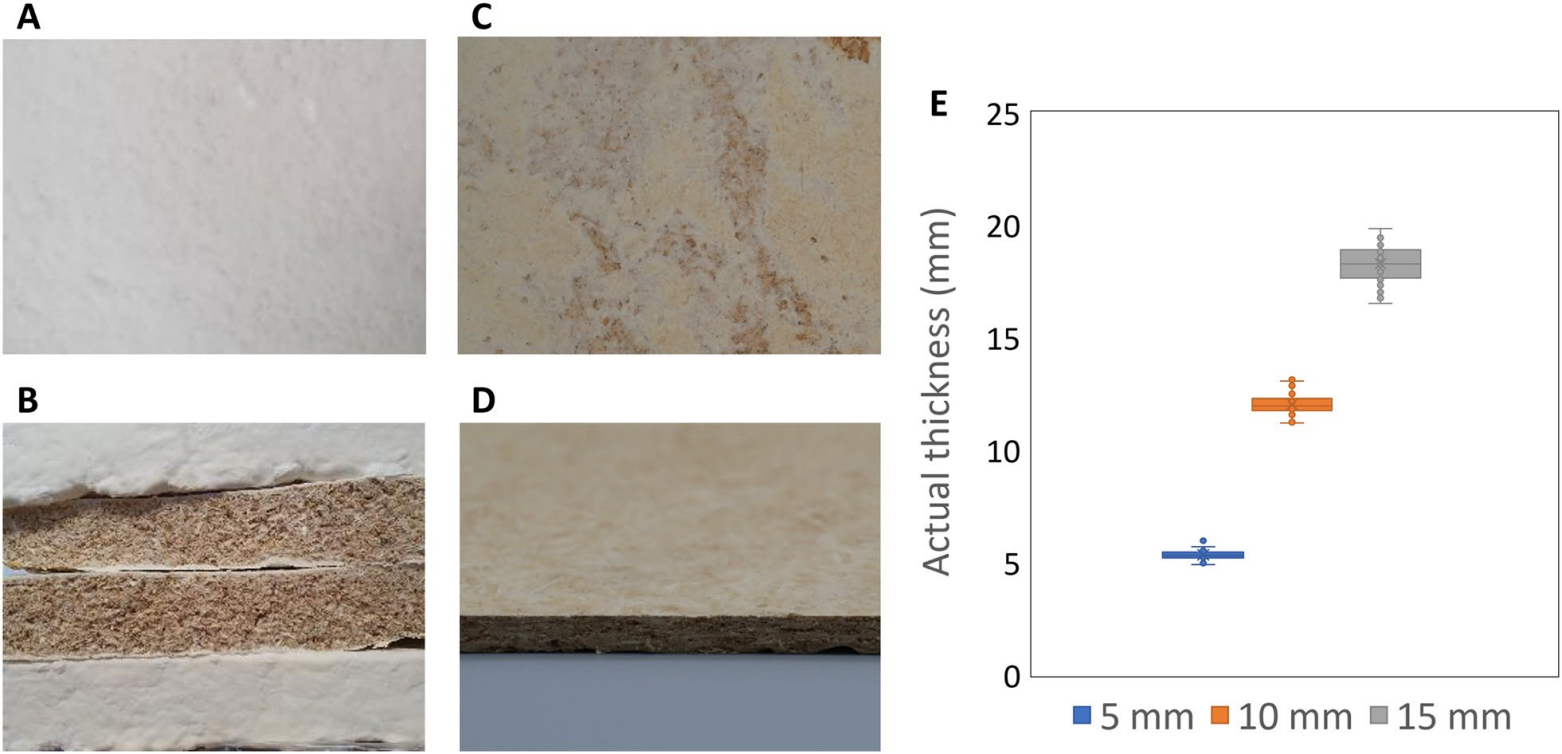
Fungal-based composite made of *F. fomentarius* and hemp shives prior and post heat-pressing. **A** Composite appearance after 14 d of cultivation prior to drying. An evenly whitish-coloured coat defined by the aerial mycelium of *F. fomentarius* gives the composite surface a fluffy texture. **B** Cross-section of 5 cm composites after drying. **C**, **D** Top view and cross section of a board composite with a target thickness of 5 mm that was heat-pressed at 140 °C. Note the subtle brown colorization of the board surface. **E** Actual thicknesses of the boards after heat-pressing, which deviate from the target thicknesses of 5, 10 and 15 mm

In a screening effort, different temperatures (20–220 °C) and pressing times (30 s, 3 min and 6 min) were tested for heat-pressing of *F. fomentarius* composites with an original thickness of 5 cm towards a final thickness of 10 mm. Basically, low temperatures or too short pressing times resulted in boards which did not stay compressed. Below 60 °C, the fungal composites were mechanically crushed and lost their material cohesion (data not shown). Pressing between 80 °C for 6 min or 120 °C for 30 s resulted in some binding of hemp shives with the mycelium, but not enough to consolidate them with the required stability. At 160 °C onwards, however, stable mycelium-bonded boards were obtained (Fig. 3, Additional file 1: Fig S1, and data not shown). Interestingly, 160 °C was also the temperature where we noticed a slight browning of the surface of *F. fomentarius* composites (Fig. 3), accompanied with a smell reminding that of baked goods right after the pressing process. This phenomenon could be explained by the Maillard reaction, which refers to the cross-linking of amino acids and sugars at temperatures above 140 °C and being responsible for the brown colouring during the baking process [21]. Similar browning during heat-pressing of fungal-based composites has already previously been reported for *Pleurotus* and *Trametes* species in combination with straw or sawdust, whereby the amino acids and sugars may stem from hyphal surfaces and/or the cell wall of the hemp shives [18]. As 160 °C was the lowest temperature that we were able to obtain boards that were firm, stiff, and rigid, we decided to continue with this temperature. In doing so, we produced heat-pressed boards with a target thickness of 5 mm, 10 mm or 15 mm (see Materials and Methods).

**Fig. 4:**
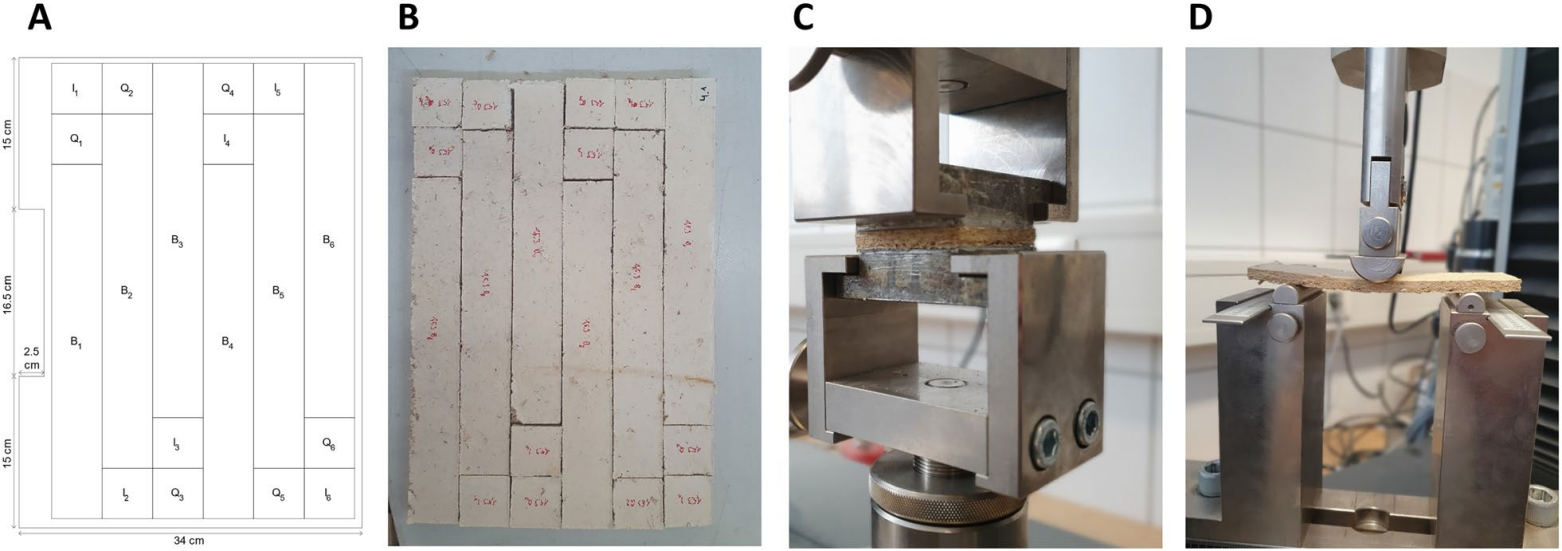
Experimental setup for material testing according to norm EN 312. **A**, **B** Cutting pattern and exemplar specimen of a *F. fomentarius* heat-pressed particleboards with 15 mm target thickness. **C** Apparatus for determining transverse tensile strength. **D** Apparatus for determining flexural strength

All three particleboard types were subjected to material testing according to the requirements for interior use at dry spaces, which are defined in the norm DIN EN 312 (Type P1, plates in dry areas; 2010). According to this norm, testing of density, flexural strength, transverse tensile strength, modulus of elasticity, and water absorption is required. Six specimens for each particleboard type were cut into smaller pieces of predefined size according to the norm EN 312 (Fig. 4, Additional file 1: Fig S2). The determination of the densities of all specimens showed they scatter about 20% within each particleboard with highest values in its middle region (Additional file 1: Fig S3). On average, particleboards with a target thickness of 15 mm/10 mm/5 mm displayed a density of 188 kg m^−3^/286 kg m^−3^/666 kg m^−3^, respectively (Additional file 1: Fig S3). The comparatively large scattering of the density values of the plate samples is mainly caused by the manual dispersion of substrate particles to produce the blank. The differences are multiplied through density changes resulting from pressing.

**Fig. 5:**
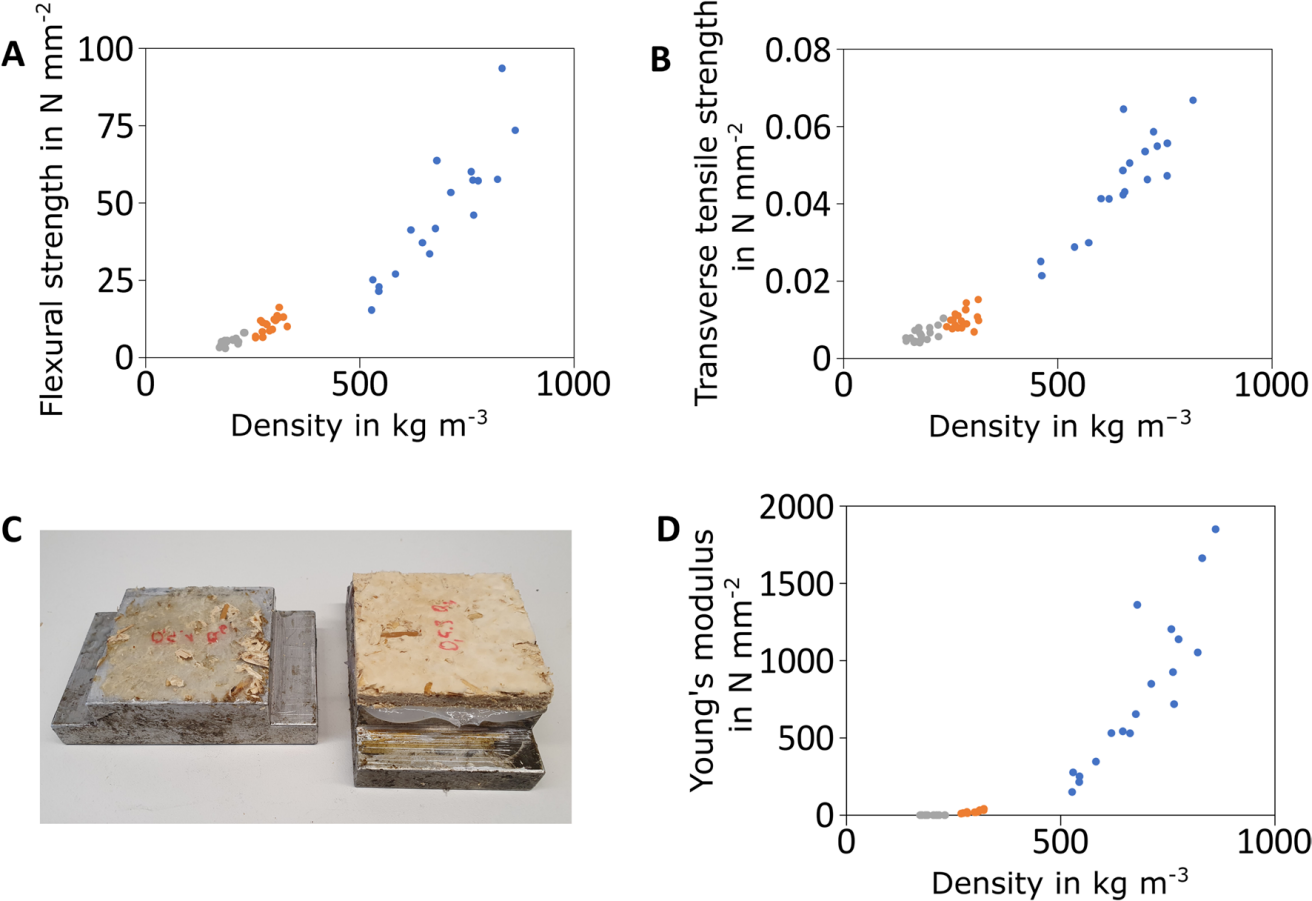
Mechanical properties of heat-pressed particleboards made of *F. fomentarius* and hemp shives. **A** Flexural strength as a function of density; **B** Transverse tensile strength as a function of density; **C** Example for a cover layer from a 5 mm specimen that has been torn off the board; **D** Young`s modulus as a function of density. Data for 5, 10, 15 mm boards are given in blue, orange, and grey, respectively

Data for flexural strength, transverse tensile strength, and modulus of elasticity in relation to the density of each specimen are summarized in Fig. 5. Clearly, these parameters increase with lower thickness and thus higher densities of the particleboards. This strongly suggests that the manual manufacturing and heat-pressing process of *F. fomentarius* composites need to be better controlled to ensure less variation in density (e.g., use of substrate particles with more uniform shapes and sizes, avoidance of agglomerates, automation of the manufacturing process). With respect to flexural strength, particleboards with a target thickness of 5 mm achieved a maximum value of 10.06 N mm^−2^, which nearly meets the standard of 11.5 N mm^−2^ for particleboards with a thickness of 3–6 mm (DIN EN 312 Type P1, 2010). However, a flexural strength of at least 10 N mm^−2^ is required for 13–20 mm thick boards (DIN, 2010), which was not achieved with the particleboards tested (Fig. 5A).

The DIN EN 312 for Type P1 requires a transverse tensile strength of 0.31 N mm^−2^ when the particleboard is 3–6 mm thick and at least 0.24 N mm^−2^ for 13–20 mm thick particleboards. The particleboards obtained with *F. fomentarius* and hemp shives did not meet these requirements (Fig. 5B). The average transverse tensile strength of particleboards with a target thickness of 5 mm (10 mm) reached only about 12% (3%) of the predefined threshold. We assume however, that the transverse tensile strength of the 5 mm specimens was measured insufficiently. The heat-pressing process forms a paper-like cover layer from the aerial mycelium of the blank. This represents the boundary layer of the board specimen adhered to the specimen holder. Since its adhesive properties to the board are lower than the transverse tensile strength of the remaining plate cross-section, only the cover layer is torn off the board (Fig. 5C). Thus, the data presented in Fig. 5B for 5 mm boards rather represent the adhesive strength of the surface layer to the core of the particleboard instead of the board itself. The problem could likely be avoided if the surface of the board samples would be calibrated by sanding, as is done, for example, in the production process in the wood-based materials industry. We therefore conclude that particleboards of 5 mm target thickness have higher transverse tensile strengths than summarized in Fig. 5B. This is also evident from the results for the specimens with thicknesses of 10 and 15 mm, but lower densities in this case. Here, the force ratios from the top layer to the board body are reversed compared to the rest of the specimen cross-section.

Figure 5D depicts values that were determined for the modulus of elasticity (Young`s modulus) dependent on the specimen’s density for particleboards with a target thickness of 5 mm and 10 mm. Whereas 10 mm particleboards displayed an Young`s modulus of about 10–40 N mm^−2^, 5 mm particleboards achieved higher values between 150 and 1850 N mm^−2^. Notably, an Young`s modulus is not demanded after the DIN EN 312 Type P1 regulation, but particleboards of the DIN EN 312 Type P2 must have an Young`s modulus of 1950 N mm^−2^ (Type P2 refers to plates in dry area including furniture). Only one of the 5 mm particleboard specimen peaked at 1850 N mm^−2^ and nearly met this requirement, suggesting that the composite density before pressing needs to be better controlled to limit heterogeneity. Further, achieving a higher density can also lead to a higher Young’s modulus of the composite materials as shown in a recent study testing materials made of *Pleurotus eryngii*, hardwood and coffee grounds [22].

**Fig. 6:**
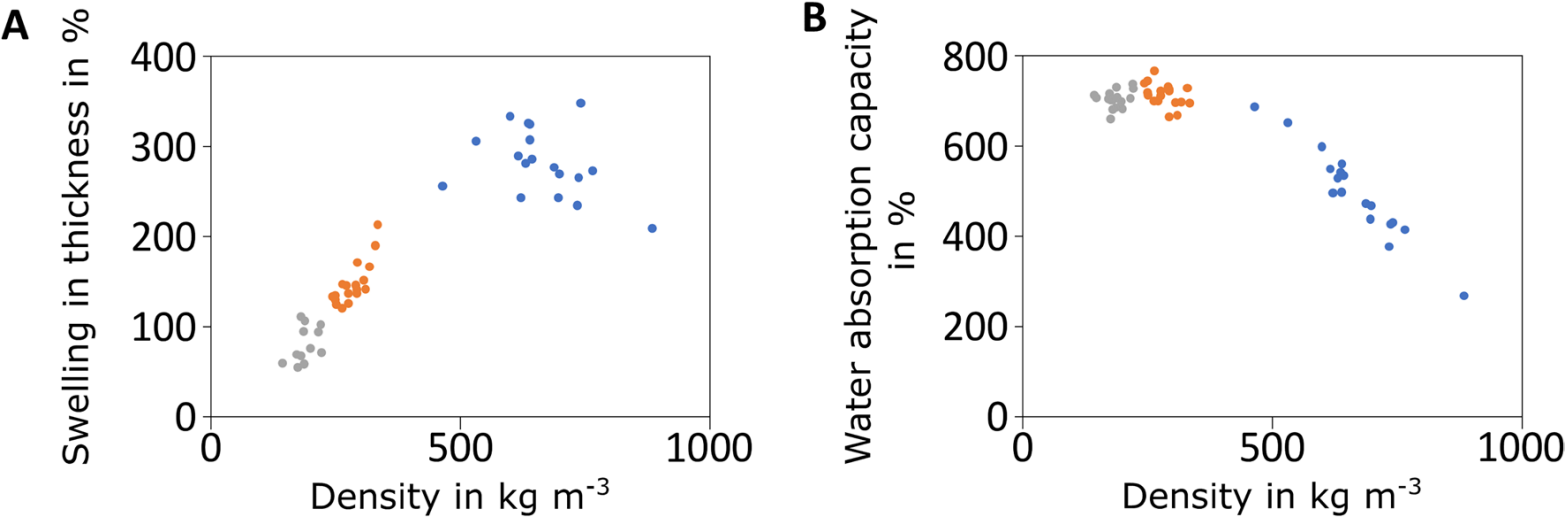
Physical properties of heat-pressed particleboards made of *F. fomentarius* and hemp shives. **A** Swelling in thickness as a function of density; **B** Water absorption capacity as a function of density. Data for 5, 10, 15 mm boards are given in blue, orange, and grey, respectively

According to DIN EN 312, there are no general requirements regarding water uptake and thus swelling in thickness of Type P1 boards (DIN, 2010). As summarized in Fig. 6, particleboards of *F. fomentarius* and hemp shives exert a high water absorption and swelling capacity when placed in a water bath for 24 h. As expected, particleboards of larger target thickness (15 mm, 10 mm) swell less strongly than 5 mm particleboards because of less heat-pressing (Fig. 6A). Interestingly, water absorption capacity showed little correlation with the density of the specimen and varied between 650 and 760% for 10 mm and 15 mm particleboards, respectively. Less water was taken up by particleboards of 5 mm thickness; however, the values averaged between 370 and 550%. Future analyses could potentially reveal whether the bonding mechanism between *F. fomentarius* hyphae and hemp shives (which supposedly is dependent on the strength of the heat-pressing force) affect water absorption capacity of the particleboards.

### Microstructures of uncompressed and compressed *F. fomentarius* composites

**Fig. 7:**
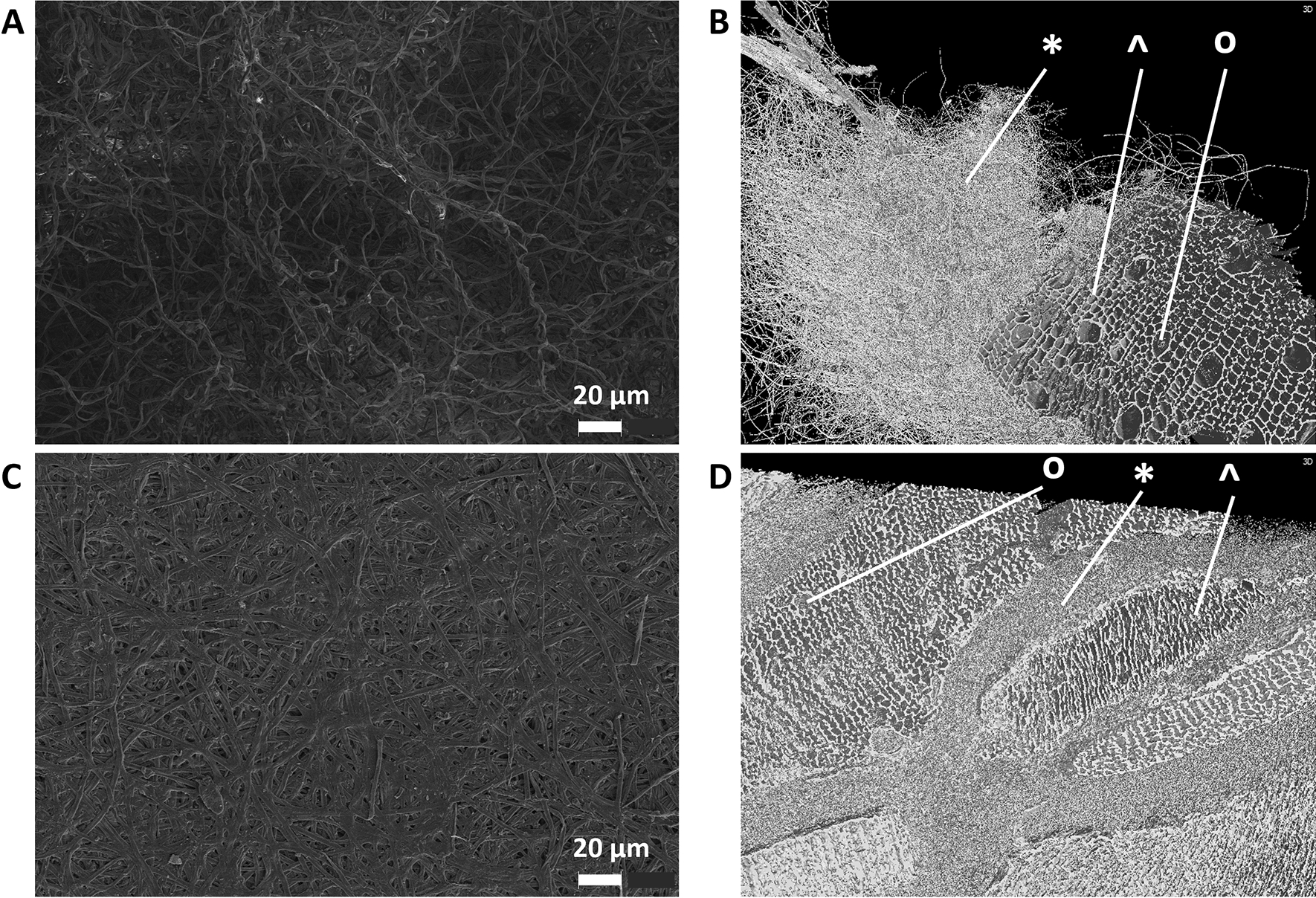
SEM and µCT images of composites made of *F. fomentarius* and hemp shives. **A** The surface of an uncompressed composite analysed via SEM, Bar, 20 µm; **B** Centre piece of an uncompressed composite analysed via µCT; **C** The surface of a heat-pressed particleboard with 5 mm target thickness, Bar, 20 µm; **D**, Centre piece of a heat-pressed particleboard with 5 mm target thickness analysed via µCT **B**, **D** * mycelium, o hemp xylem, Λ hemp pore

With scanning electron microscopy (SEM) and X-ray micro-computed tomography (µCT), we analysed microstructures of the composites that were obtained for *F. fomentarius* during growth on hemp shives before and after heat-pressing. Figure 7A highlights SEM images that mirror the three-dimensional, intertwined and fluffy appearance of the aerial mycelium of *F. fomentarius* before heat-pressing. After heat-pressing, however, the aerial mycelium becomes completely flattened and forms a two-dimensional layer rather than a voluminous network (Fig. 7C). Hence, the foam-like and elastic surface of *F. fomentarius* composites becomes condensed into a thin sheet of mycelium through heat-pressing, resembling a “paper coating” on the particleboards. Figure 7B and D compare the microstructures before and after heat-pressing using µCT imaging with a focus on the inner parts of the composites. Due to a better resolution compared to SEM, hemp shives with small and large pores (xylems) are well recognizable which are surrounded by a large network of *F. fomentarius* hyphae (Fig. 7B). Heat-pressing clearly compressed the hemp shives, whereby especially the xylem vessels became deformed and had smaller void spaces. Also, the hyphal network appeared to be pressed between hemp shives to form tighter and denser connections. (Fig. 7D).

**Fig. 8:**
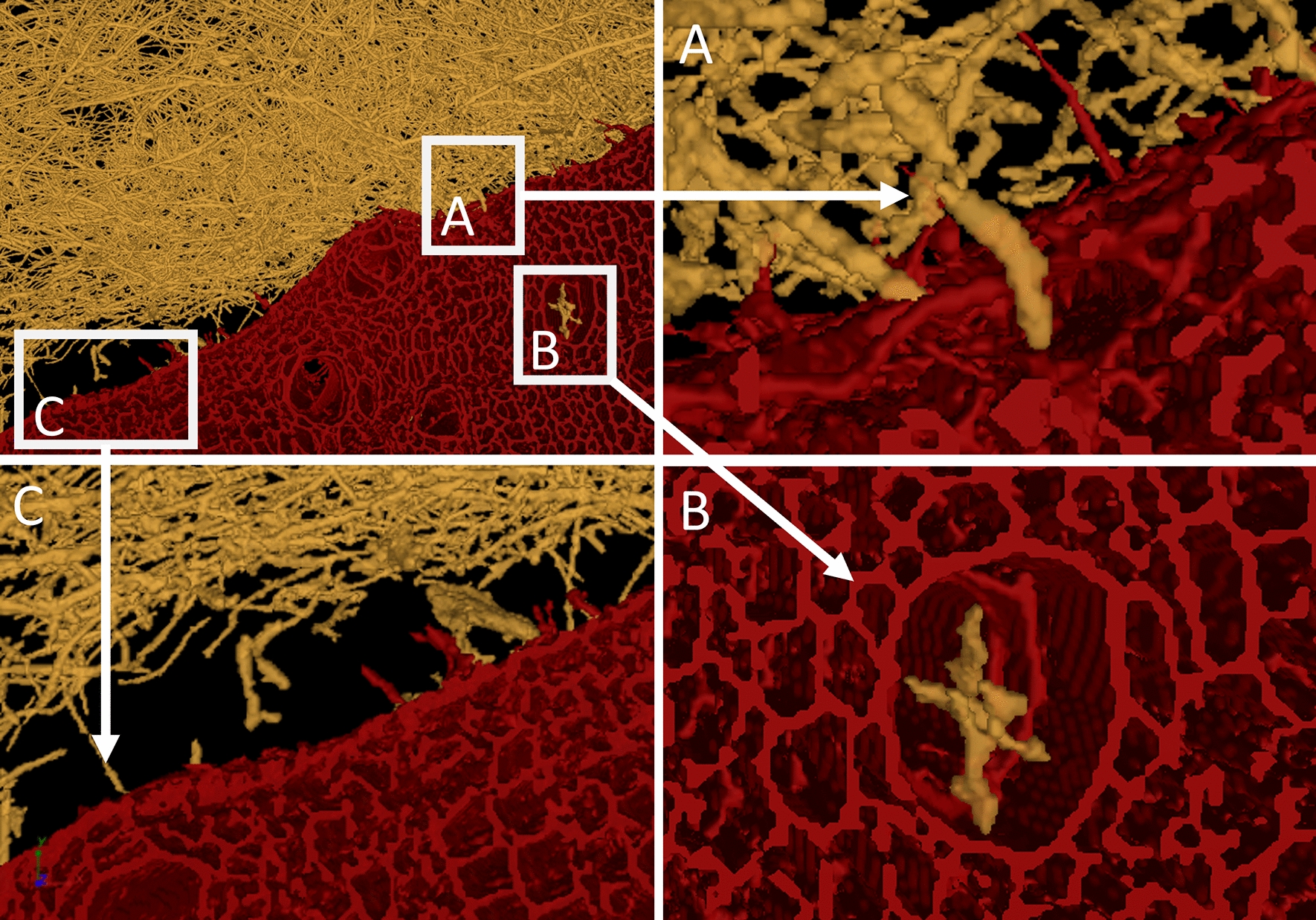
The processed µCT images of composites made of *F. fomentarius* and hemp shives. *F. fomentarius* is shown in yellow and the hemp shives in red. The subpanels A-B show enlargements of the marked regions. Hyphae attaching to the hemp shive substrate (**A**) and hyphae within xylem vessels (**B**) are depicted. Note that the outermost layer of the hemp shive appears thicker than the inner cell, which could potentially hint at an ongoing decomposition process of lignin and (hemi-)cellulose, which remains to be shown **C**

Figure 8 highlights that hyphae of *F. fomentarius* make contact with hemp shives particles and also grow inside xylem vessels. However, due to the still limited resolution of µCT and similar density between hyphae and hemp shives, it was not possible to determine whether hyphae grew inside the xylems through existing holes or penetrated their cell wall through decomposition. Reconstructed 3D videos that visualize growth of *F. fomentarius* hyphae on and penetration into a hemp shive before pressing and compression of both after heat-pressing are depicted in Additional file 1: Fig S5 and Additional file 1: Fig S6, respectively. µCT images of unpressed *F. fomentarius* composites where hemp shives and hyphae were segmented are shown in Additional file 1: Fig S7.

**Fig. 9:**
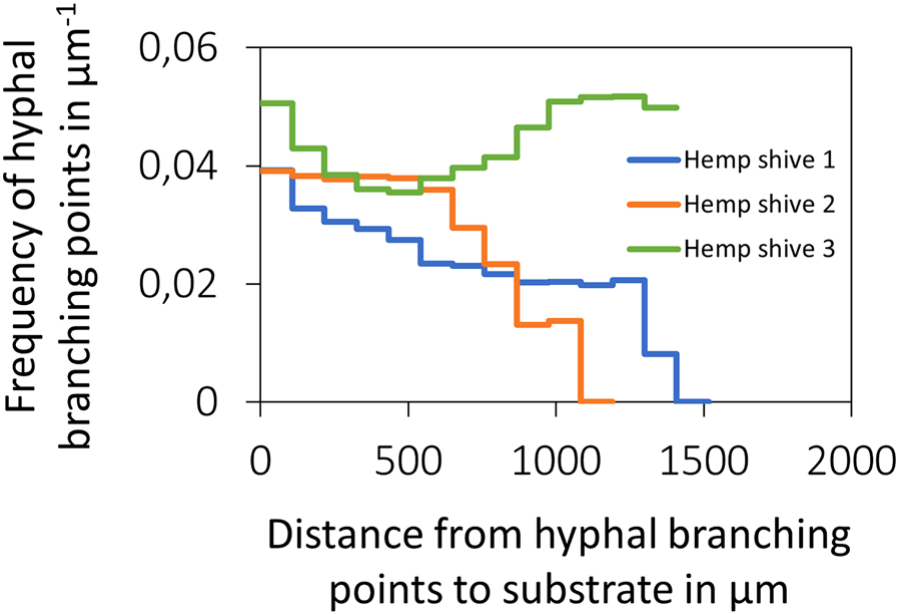
Hyphal branching frequency depends on the distance of the hyphae to the surface of hemp shives

Remarkably, the branching frequency of *F. fomentarius* hyphae depends on the distance of the mycelium to the surface of hemp shives. As quantified in Fig. 9, the relative frequency of branching points increases with greater proximity to the hemp shive substrate as shown for hemp shive 1 and 2, implying that nutrient sensing by *F. fomentarius* could potentially induce hyperbranching and thus formation of a denser hyphal network for faster nutrient acquisition, a hypothesis that remains to be verified in future studies. For hemp shive 3, however, the hyphal branching frequency decreases till a distance of 400 µm and starts increasing again from 500 µm onwards until it reaches a similar value as the initial one at a distance of 1200 µm. We assume that the higher branching frequency and thus denser hyphal network in the outer region of hemp shive 3 (see Additional file 1: Fig S7 and Additional file 1: Fig S8) can likely be attributed to the presence of another hemp shive that was positioned close to the outer dense hyphal network but became unintentionally removed during the sampling process.

Based on CT image data of uncompressed composites, we were also able to compute an average hyphal diameter of 2.88 ± 0.07 µm for *F. fomentarius* when feeding on hemp shives, which has the similar value as we have determined earlier for growth on *F. fomentarius* in liquid medium by light microscopy [10]. In addition, we calculated a solid fraction of hemp shives that ranges from 0.22 to 0.26. Based on a solid density of 1450 $\mathrm{kg }\,{m}^{-3}$ [23], the apparent density of hemp shives determined by CT image analysis ranges from 319 $\mathrm{kg }\,{\mathrm{m}}^{-3}$ to 363 $\mathrm{kg }\,{\mathrm{m}}^{-3}$, which is close to what has been reported earlier [24]. The increase in the solid fraction of hemp shives caused by heat-pressing was evaluated for samples from boards with 5 mm target thickness. Here, we could observe different increase over the cross-section with a solid fraction of shives of 0.45 on the surface layer and of 0.50 in the middle layer, respectively.

## Conclusions

Approaches to significantly reduce global CO_2_ emissions are pivotal to mitigate climate change. Central to this is the transformation of the built environment, as it contributes to 40% of anthropogenic CO_2_ emission. The problem becomes exacerbated due to urban growth, which is expected to double the global floor area by 2060 [25]. To achieve climate neutrality (i.e., the Paris Agreement of 1.5 degrees by 2030), fundamentally new concepts of circularity need to become implemented by the building sector. In this study, we thus investigated several properties of composite materials consisting of mycelia of the basidiomycete *F. fomentarius* and hemp shives to evaluate their potential as building materials for construction or insulation purposes. To allow data comparison with already existing fossil-based or renewable construction materials, we followed EN standard testing procedures.

**Fig. 10:**
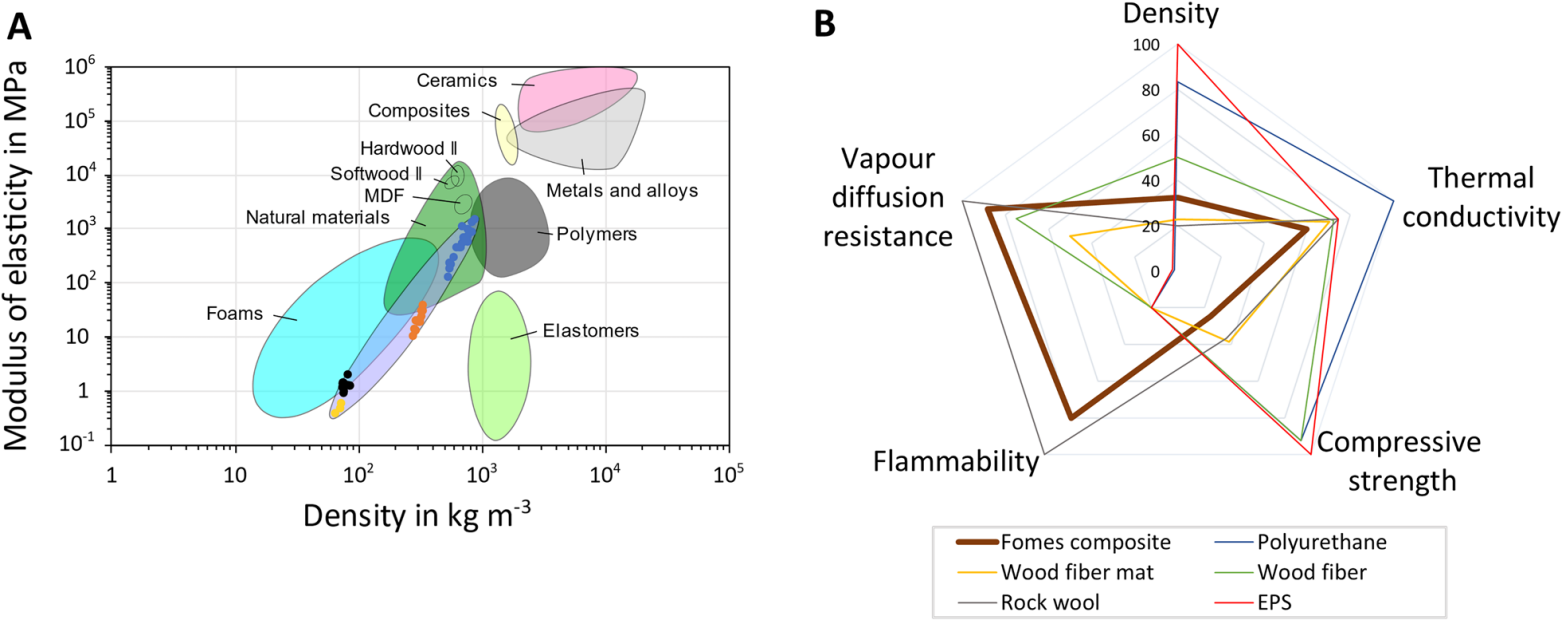
Comparison of composites made of *F. fomentarius* and hemp shives with different approved construction materials that are most frequently used in Germany. **A** Ashby diagram highlighting elasticity as a function of density. Dots correspond to the values obtained in this study or published earlier [10]. Black, uncompressed composites this study; yellow, uncompressed composites [10]; orange, 10 mm compressed composites; blue, 5 mm compressed composites. **B** Radar chart representing standardized values for different materials. The higher the value the better the performance on a scale between 0 and 100%. Raw data used for standardization were taken from this study and from data sheets of industrially used materials (Additional file 1: Fig S4)

Figure 10 highlights six out of twelve parameters that we have determined in this study and compares them with values of established fossil-based and renewable construction matrials. An Ashby map for the modulus of elasticity as a function of composite density (Fig. 10A) shows that uncompressed and heat-pressed *Fomes*-hemp composites form their own material class, which shares properties with foams and natural (wood) materials. This is in agreement with data obtained for other fungal species and plant-based substrates [2, 6, 26]. A radar chart for five parameters important for thermal insulation is depicted in Fig. 10B, where *Fomes*-hemp composites are compared with approved insulation materials including plastic-based (polyurethane, EPS), rook wool, and wood fibres. It can be concluded that *Fomes*-hemp composites are suitable for thermal insulation. In contrast to diffusion-tight plastic-based insulation materials, composites of *F. fomentarius* and hemp shives show very good water vapour permeability values. Nevertheless, they exert a lower compressive strength and their insulation efficiency (i.e. thermal conductivity) is lower than EPS and thus needs improvement in future optimization efforts (Fig. 10B). Encouragingly and in contrast to plastic-based materials, *F. fomentarius* composites display flame-retardant properties with moderate smoke development only. Still, smoke composition needs to be determined to prove that thermal degradation processes of hyphal and hemp components do not lead to the release of harmful halogenated volatile molecules. Respective analyses are thus currently ongoing in our lab (manuscript in preparation).

Further efforts have been made in this study to produce mechanically resilient materials through heat-pressing. Due to the manual manufacturing process, some results for the test specimens scattered considerably. Even if individual variants already show promising properties, the majority of the values determined for compressive strength, for example, are too low. Hence, future optimisation efforts shall include standardisation of the cultivation process as well as improvement of process parameters (e.g. temperature and duration during heat-pressing). Importantly, combined sandwich models, e.g. unpressed composites surrounded by pressed particleboards or wood panels, or alternatively, reinforced grid structures obtained via 3D printing of bioplastics or recycled concrete could be viable approaches to increase load-bearing capacities of *Fomes* composites [27].

Most importantly, the production of fungal composites must become scalable from laboratory to industrial conditions to achieve market introduction. To minimise the natural variability, this also includes factors in the supply chain of the input materials to standardise the substrate density, e.g. by pre-compression and weighing of the filled moulds. Even with non-compacted building materials such as insulation materials, the material density as well as its distribution in the material has a decisive influence on the resulting properties. This also applies to compacted material panels if they are to meet static requirements. Here, corresponding improvements of the manufacturing process must be sought in the future.

## Methods

### Cultivation of *F. fomentarius* and manufacturing of composite materials

*F. fomentarius* strain PaPF11 from the Berlin-Brandenburg area (Germany) was established earlier as host for composite production [10]. All experimental details on the manual manufacturing process can be obtained from this resource [10]. For generation of specimens for thermal conductivity testing, aluminium sheets were placed on top of the main cultures to obtain smooth composite surfaces. When specimen of different heights were produced (5, 6, 8, and 10 cm), specimens with a height of 8 or 10 cm were dried for 96 h instead of 48 h at 50 °C. For all cultivations, hemp shives from Hempparade (purchased from Futtermittel Louven e.K., Mönchengladbach, Germany, produced by HempFlax Group B.V., Oude Pekela, The Netherlands) were used as substrate. For minimum to maximum Feret diameters of hemp shives, see Additional file 1: Fig S9.

### Compression testing of *F. fomentarius* composites

Cuboid specimens (at least four biological replicates for each height) were produced according to the protocol published earlier [10]. Compression testing was performed with varying crosshead speed depending on the thickness of the specimens (5 cm specimen with 5 mm min^−1^, 6 cm specimen with 2 mm min^−1^, 8 cm specimen with 8 mm min^−1^, 10 cm specimen with 9.5 mm min^−1^) and a preload of 20N. Stress at 10% compression was measured according to DIN EN 826 using a compression testing machine. Parallelism and evenness of the specimen between the compression plates did not allow to exceed a limit deviation of 0.5% of the edge length. Before measurements, specimens were acclimatized for 6 h at 23 °C ± 5 °C in a laboratory environment without controlled humidity and measured at this temperature. Immediately after the test, the height of the samples was determined. Stress–strain curves were evaluated to calculate stress at 10% compression (δ_10_):

$${\delta }_{m}= {10}^{3}\frac{{F}_{m}}{{A}_{0}} [kPa]$$

*F*_*m*_  = maximum strength [N]

*A*_*0*_  = cross section [mm^2^]

*δ*_*m*_ = stress

$${\upvarepsilon }_{m}= \frac{{X}_{m}}{{d}_{0}}100 [\%]$$

*X*_*m*_  = strain at *F*_*m*_ [mm]

*d*_*0*_ = initial thickness [mm]

*ε*_*m*_ = strain

### Determination of thermal insulation conductivity

The thermal insulation conductivity (λ) was quantified with a two-plate device (HFM300, Linseis) consisting of a heating and cooling plate, whereby a constant and steady heat flux (ϕ) on the even and parallel surface of the specimen was produced. Generally, the instrument consisted of a homogenous carrier, a temperature sensor to measure differences on the surface and several temperature sensors to determine the surface temperature. Before testing the specimen, an additional drying at 105 °C was carried out until the mass constancy was reached and the specimen stored at standard climate (23 °C and 65% relative humidity) for 4 days. The thermal flux (ϕ) and the temperature difference between the surfaces of the test plates (ΔT) were measured to determine the thermal insulation conductivity (λ) according to the following formula:

$$\lambda  = \frac{{\phi d}}{{A\left( {T_{1}  - T_{2} } \right)}}\,\left[ {\frac{W}{{m~K}}} \right]$$

*ϕ* = power supply of heat flux to heating plate [W]

*T*_1_ = average temperature on warm side of the specimen [K]

*T*_2_ = average temperature on cold side of the specimen [K]

*A* = measuring area [m^2^]

*d* = thickness of the specimen [m]

### Determination of water vapour transmission

The water vapour transmission coefficient was determined according to DIN EN 12086. Here, the thermal transmission from one fluid/gas to the other through a solid specimen (due to the temperature differences between the fluids/gases becomes) is measured. Therefore, the specimen was installed in a test vessel, which was open on top, filled with a desiccant, and laterally sealed. The measurement was carried out at a standard climate of 23 °C and 65% relative humidity. Differences between the outer climate and vessel regarding the vapour pressure, causes a vapour-flow through the solid specimen. By multiple weight measurements of the installation with an analytical balance, the vapour diffusion current density (g) was calculated at consistent mass change (steady state). Before the experiment was carried out, specimens were acclimatized for 6 h at 23 °C ± 5 °C and 65% relative humidity. Mass change–time diagrams were recorded for the subsequent calculations and the following variables determined according to the equations given below: periodic mass change (G), vapour diffusion current density (g), vapour diffusion transmittance (W), vapour diffusion conductivity coefficient (δ), vapour diffusion resistance coefficient (µ):

$$g=\frac{G}{A} \left[kg\, {m}^{-2 }\,{s}^{-1}\right]$$

G = periodic mass change (m_2_-m_1_/t_2_-t_1_) [g s^−1^]

*A* = test surface of specimen [m^2^]

$$ W = \frac{G}{{A \cdot \Delta p}}\left[ {kgm^{{ - 2}} \,s^{{ - 1}}\, Pa^{{ - 1}} } \right] $$

*Δp* = partial pressure difference of vapour [Pa]

A = 23 °C, rel. humidity 50% ± 3% (moist state) → 1400 Pa

B = 23 °C, rel. humidity 85% ± 3% (moist state) → 2390 Pa

C = 23 °C, rel. humidity 93% ± 3% (moist state), 50% ± 3% (dry state) → 1210 Pa

$$ \delta  = W \cdot d\left[ {kg\,m^{{ - 1}} \,s^{{ - 1}}\, Pa^{{ - 1}} } \right] $$

*d* = thickness of specimen [m]

$$\mu =\frac{{\delta }_{air}}{\delta } [-]$$

*δ*_*air*_ = average air pressure

### Flammability testing

Fire classification of a *F. fomentarius* composite was performed according to the norm DIN EN 13501–1. Two specimens were tested by perpendicularly clamping them into the installation and central ignition. The flammability was visually documented within 30 s. Smoke development was measured according to EN 13823 and the smoke growth rate determined in m^2^ s^−2^, which was used for the classification into s1, s2 or s3.

### Heat-pressing

Composites of *F. fomentarius* and hemp shives with a height of 5 cm were heat-pressed after drying in a one-step process with semi-industrial heat-press Rucks KV 243 (Rucks Maschinenbau, Glauchau, Germany). The heat press was adjusted to a temperature of 20–220 °C and the pressing time was set up to 6 min. The thickness and thereby the density of the produced composite boards was ensured by merging the heated press jaws together against the metal spacers (Additional file 1: Fig S1A and B). Three different thicknesses (5 mm, 10 mm, and 15 mm) were produced at 160 °C for 180 s in biological triplicates and used for material testing.

### Material testing of *F. fomentarius* particleboards

Material testing was performed according to the requirements for interior use at dry spaces and the respective EN norm (EN 312, 2010), using a material testing machine Zwick Z010 (Zwick-Roell, Ulm, Germany). Tables 1 and 2 summarize the properties analyzed and the sizes of the test specimen.

**Table 1:** Mechanical and physical properties determined for *F. fomentarius* boards

Property	Norm	Designation	Symbol	Unit
Density	EN 323	I/Q/B	$\rho$	${kg\, m}^{-3}$
Swelling in thickness	EN 317	I	${G}_{t}$	%
Water absorption capacity	EN 317	I	${W}_{t}$	%
Transverse tensile strength	EN 319	Q	${f}_{t}$	${N \, mm}^{-2}$
Flexural strength	EN 310	B	${f}_{m}$	${N \, mm}^{-2}$
Modulus of elasticity (Young`s modulus)	EN 310	B	${E}_{M}$	${N \, mm}^{-2}$

**Table 2:** Sizes of the test specimen tested

Designation	Length (a) x width (b) in [mm]	Replicates
I	50 × 50	3 × 6
Q	50 × 50	3 × 6
B	150 × 50 250 × 50 350 × 50	3 × 6

The length $a$ of the specimens for the flexural strength and modulus of elasticity depends on the board thickness (EN 310, 1993) and can be calculated according to the following equation:

$$a=20*{t}_{N}+50\, [mm]$$

t_N_ = nominal thickness [mm]

Thus, three different cut schemes were followed according to the norm of sampling, cutting, and monitoring (DIN, 1994). The cut schemes of each board type are summarized in Additional file 1: Fig S2. Note that heat-pressing leads to an increase of the board size due to flattening, hence the boards were trimmed along the edges accordingly. Six specimens for each material test (Table 1) were cut using a circular saw (SC3 W, Mini Max, Italy) equipped with a 3.2 mm circular saw blade (Z28, H.O. Schumacher + Sohn, Germany). The particle boards were considered isotropic, which is why specimen ${B}_{"n"}$ were extracted from just one board axis. The specimens were labelled and stored in a climate chamber (WK11-600/40, WEISS) at norm climate (20 °C, 65% humidity) overnight before measurements. The size and mass of all specimens were determined with a digital caliper (PS 7215, Burg Wächter, Germany) and a scale (TE3102S, Sartorius, Germany), respectively.

Raw density ρ of each specimen was determined according to norm EN 323 (1993):

$$\rho =\frac{m}{a*b*t}*{10}^{6} \left[kg\, {m}^{-3}\right]$$

$m$ = mass of specimen [g]

$t$ = thickness of specimen [mm]

$a;b=$ length; width of specimen [mm]

Swelling in thickness and water absorption capacity refers to the expansion of a specimen after being immersed in a water bath (GFL 1004, GFL) at 20 °C for 24 h, after which the size and mass of a specimen were again determined. The swelling thickness ${G}_{t}$ and the water absorption capacity ${W}_{t}$ were calculated according to the norm for fiber and particleboards (DIN, 1993c) as follows:

$${G}_{t}=\frac{{t}_{2}-{t}_{1}}{{t}_{1}}*100\, [\%]$$

${t}_{1}$ = thickness of specimen before immersion [mm]

${t}_{2}$ = thickness if specimen after immersion [mm]

$${W}_{t}=\frac{{m}_{2}-{m}_{1}}{{m}_{1}}*100\, [\%]$$

${m}_{1}$ = mass of the specimen before immersion [g]

${m}_{2}$ = mass of specimen after immersion [g]

The transverse tensile strength of the particleboards was determined by gluing the specimens to metal test yokes using a glue gun. After the yokes were clamped into the sample holder, they were pulled apart by a material test machine (Z010, Zwick/Roell, Germany) until the specimen ripped off (Fig. 4C). The transverse tensile strength ${f}_{t}$ was calculated according to the test norm for fibre particle and boards (DIN, 1993b) as follows:

$${f}_{t}=\frac{{F}_{max}}{a*b} [N \,{mm}^{-2}]$$

$${F}_{max}$$ = breaking force [N]

a; b = length; width of the specimen [mm]

For determining the flexural strength ${f}_{m}$, the specimens were placed onto two bearings. The distance of the bearings was determined according to the norm for wood materials (DIN, 1993a):

$${l}_{1}={l}_{2}-50 \,[mm]$$

${l}_{1}$ = distance between the centers of the bearings [mm]

${l}_{2}$ = length of the specimen [mm]

A load head was moved downwards by the test machine until the specimen broke (Fig. 4D). The distance between the bearings ${l}_{1}$ was calculated with:

$${l}_{1}=20*{t}_{N} [mm]$$

${t}_{N}$ = nominal board thickness [mm]

The flexural strength ${f}_{m}$ was calculated according to the following equation:

$${f}_{m}=\frac{3{*F}_{max}*{l}_{1}}{2*b*{t}^{2}} \left[N\,{ mm}^{-2}\right]$$

$${F}_{max}$$ = breaking force [N]

${l}_{1}$ = distance between the centers of the bearings [mm]

$b$ = width of the specimen [mm]

$t$ = thickness of the specimen [mm]

The modulus of elasticity ${E}_{M}$ (Young`s modulus) was determined according to the wood materials norm (DIN, 1993a) and the following equation:

$${E}_{M}=\frac{{l}_{1}^{3}*({F}_{2}-{F}_{1})}{4*b*{t}^{3}({a}_{2}-{a}_{1})} [N\, {mm}^{-2}]$$

${l}_{1}$ = distance between the centers of the bearings [mm]

$b$ = width of the specimen [mm]

$t$ = thickness of the specimen [mm]

${(F}_{2}-{F}_{1})$ = increase of the force in the rectilinear range of the force–deflection diagram [N]; ${F}_{1}$ has to be approximately 10% and ${F}_{2}$ approximately 40% of the breaking force (${F}_{\mathrm{max}}$)

${(a}_{2}-{a}_{1)}$ = increase of the deflection at the center of the specimen (according to ${F}_{2}-{F}_{1}$) [mm]

### Microstructural characterisation

Scanning electron microscopy (SEM, CamScan Series 2, Obducat, Sweden) was performed to analyse hyphal growth of *F. fomentarius* on lignocellulosic substrates. In brief, SEM was used in the high vacuum, secondary electron mode with an accelerating voltage of 14 kV. The specimen was gold sputter coated (Cressington Sputter Coater, 108 Auto, Tescan GmbH, Dortmund, Germany) at 30 mA for 40 s.

X-ray micro-computed tomography (µCT) was conducted at the DESY (Deutsches Elektronen-Synchrotron), Hamburg. Ten samples each of a volume of about 4 mm^3^ were extracted with a scalpel and tweezers from different parts of unpressed and heat-pressed *F. fomentarius* composites. The following settings were applied: Camera system: 50 MP CMOS, pixel size binned (3 × 3 binning): 1.41 μm (final voxel size), field of view with vertical image stitching: 3.71 × 4.5 mm (width x height), energy: 17.5 keV, number projections: 2401, exposure time per projection: 100 ms.

The image processing pipeline was established in Matlab (Version 2022a) and contained the following parts: (1) the segmentation of hemp shive substrate and (2) the segmentation of fungal hyphae. The preprocessing of the CT-data including the reconstruction of the 3D images and removal of the sample holder based on the methods described in Müller et al. 2023 [28]). Before the segmentation of hemp shives (1), a non-local-mean filter was used (MATLAB function imfiltnlm) to reduce noise in the background for each slice of the 3D image data. The sample object with substrate and fungal mycelium was binarized by using an adaptive threshold (MATLAB function imbinarize (‘adaptive’)) for the whole image. Subsequently, the binarized image was multiplied with the raw image. This step removed most of the background and the gray values of the actual sample objects (substrate and hyphae) were preserved. Thereafter, multilevel thresholds were calculated for the remaining objects with Otsu’s method (MATLAB function multithresh) to enable an accurate segmentation between hemp shives, hyphae, and noise. Since thick hyphae had similar gray values as the hemp shives, only objects larger than 140,000 µm^3^ were classified as hemp shives (MATLAB function bwareaopen). The apparent volume of the shives was determined by applying a closing operation (MATLAB function imclose) to the cross-sectional images and filling the xylem vessels (MATLAB function imfill). The size of the structuring element for the closing operation was chosen as the similar size of vessel pores. The solid fraction of the substrate ${\varepsilon }_{substrate}$ was calculated as follows:

$${\varepsilon }_{substrate}= \frac{{V}_{solid}}{{V}_{solid+pores}}$$

where ${V}_{solid}$ is the volume of the substrate without the pores and ${V}_{solid+pores}$ is the volume of the substrate including the pores.

For the segmentation of the hyphae (2), the segmented hemp shives were removed from the original gray level image, so that the hyphae and background remained on the resulting gray level image. Subsequently, a mask for the region representing only hyphae was created to exclude the background by using Otsu’s method. Small objects below 280 µm^3^ in the binarized image representing noise and small free hyphae were removed (MATLAB function bwareaopen). The hyphae in uncompressed samples were evaluated by the average hyphal diameter and relative frequency of branching points from the substrate. The average hyphal diameter ${d}_{hyphae}$ was obtained by multiplying the hyphal skeleton (MATLAB function bwskel) with the Euclidean distance transform of hyphae (MATLAB function bwdist), which assigns each hyphae pixel a value corresponding to the shortest distance to the hyphae border, and calculating the arithmetic mean of the local distances. The total hyphal length ${l}_{hyphae}$ was calculated based on the total hyphal volume ${V}_{hyphae}$:

$${l}_{hyphae}= \frac{4 {V}_{hyphae}}{\pi { {d}_{hyphae}}^{2}}$$

The number of branching points and tips of hyphae were obtained from the hyphal skeleton (MATLAB function bwmorph3). The substrate was considered as the central piece, and its surrounding volume was divided into multiple shells with a width of 50 µm. To determine the frequency of hyphal branching points within a shell, the number of branching points within this shell was divided by the total length of all hyphae in the shell.

### Supplementary Information

**Additional file 1: Fig S1**. Set-up for heat-pressing and screening approach. **Fig S2**. Cutting pattern of *F. fomentarius *heat-pressed particleboards. **Fig S3**. Density distribution of *F. fomentarius *heat-pressed particleboards. **Fig S4**. Data used for radar chart in Figure 10B. ** Fig S5**. 3D video visualizing *F. fomentarius* composites before heat-pressing.** Fig S6**. 3D video visualizing *F. fomentarius* composites after heat-pressing. **Fig S7**. Processed cross section images of unpressed *F. fomentarius* composites. **Fig S8**. Cross section image of unpressed *F. fomentarius* composite. **Fig S9**. Image analysis of hemp shives used for cultivation of *F. fomentarius*.

## Data Availability

The raw datasets generated in this study are available from the corresponding authors upon request.
